# Papillary Thyroid Carcinoma: A thorough Bioinformatic Analysis of Gene Expression and Clinical Data

**DOI:** 10.3390/genes14061250

**Published:** 2023-06-11

**Authors:** Iván Petrini, Rocío L. Cecchini, Marilina Mascaró, Ignacio Ponzoni, Jessica A. Carballido

**Affiliations:** 1 Department of Computer Science and Engineering, Universidad Nacional del Sur, Bahía Blanca 8000, Argentina; 2 Institute for Computer Science and Engineering (UNS–CONICET), Bahía Blanca 8000, Argentina; 3 Departamento de Biología, Bioquímica y Farmacia, Universidad Nacional del Sur, Bahía Blanca 8000, Argentina

**Keywords:** gene expression analysis, thyroid cancer, microarray gene expression data, RNA-seq gene expression data, GEO, TCGA, statistical learning

## Abstract

The likelihood of being diagnosed with thyroid cancer has increased in recent years; it is the fastest-expanding cancer in the United States and it has tripled in the last three decades. In particular, Papillary Thyroid Carcinoma (PTC) is the most common type of cancer affecting the thyroid. It is a slow-growing cancer and, thus, it can usually be cured. However, given the worrying increase in the diagnosis of this type of cancer, the discovery of new genetic markers for accurate treatment and prognostic is crucial. In the present study, the aim is to identify putative genes that may be specifically relevant in PTC through bioinformatic analysis of several gene expression public datasets and clinical information. Two datasets from Gene Expression Omnibus (GEO) and The Cancer Genome Atlas (TCGA) dataset were studied. Statistics and machine learning methods were sequentially employed to retrieve a final small cluster of genes of interest: *PTGFR*, *ZMAT3*, *GABRB2*, and *DPP6*. Kaplan–Meier plots were employed to assess the expression levels regarding overall survival and relapse-free survival. Furthermore, a manual bibliographic search for each gene was carried out, and a Protein–Protein Interaction (PPI) network was built to verify existing associations among them, followed by a new enrichment analysis. The results revealed that all the genes are highly relevant in the context of thyroid cancer and, more particularly interesting, *PTGFR* and *DPP6* have not yet been associated with the disease up to date, thus making them worthy of further investigation as to their relationship to PTC.

## 1. Introduction

Gene expression studies aim at understanding how the mechanisms that transcribe genes are coordinated to synthesize functional gene products, such as proteins. In particular, the analysis of gene regulation provides information on how normal cellular processes occur but also how abnormal or pathological processes are caused, for example, during the progression of several diseases such as cancer [1]. Statistical and artificial intelligence methodologies have been boosted by the accelerated development achieved by high-throughput gene expression approaches, such as microarray and RNA sequencing (RNA-seq), which are providing huge volumes of genomic data [2].

Microarray technologies are based on hybridization, while RNA-seq platforms apply new ultra-high-throughput sequencing that has only become available in recent years. Hybridization-based approaches generally require the incubation of fluorescent-labeled complementary DNA (cDNA) with predefined sequences, such as PCR products or long oligonucleotides, densely spotted on a solid modified glass surface. Unlike microarray methods, sequence-based approaches determine gene expression levels by directly sequencing cDNAs. Both technologies obtain gene expression levels by generating a relative abundance of mRNA [3].

Although the technical characteristics of the two platforms vary considerably, they both provide the possibility of obtaining lists of differentially expressed genes, where the biological interpretation of these genes plays a key role in the explanation of the experimental outputs. Moreover, the comparison of the gene list highlighted by both platforms can be useful for achieving more rigorous gene expression studies [4]. Additionally, the combination with filtering feature techniques enhances the performance of biomarker screening studies and survival analysis [5,6].

In this work, the analysis of three datasets related to thyroid cancer, which contain gene expression information, is performed. In a previous study [7], an exploratory analysis was performed on microarray gene expression datasets, comparing normal samples against Anaplastic Thyroid Carcinoma (ATC) and Papillary Thyroid Carcinoma (PTC) samples. From that investigation, HINT3 arose as a potential hub gene in ATC. In the present work, microarray and RNA-seq data are analyzed. The datasets for microarray experiments are obtained from Gene Expression Omnibus (GEO) [8,9], and the one for RNA-seq experiments from The Cancer Genome Atlas (TCGA) [10]. Another difference with a previous work is that in the present one, the aim is to discover new genes with differentiated behavior between healthy samples and PTC, leaving aside ATC samples.

The research workflow proceeds in several steps. The initial objective is to determine the degree of coincidence between the differentially expressed genes obtained from microarray data and those obtained from RNA-seq. Then, we work with a subset of selected genes and perform more punctual filtering by applying RFE (Recursive Feature Elimination). In this way, a final cluster of genes of interest is reached, their relevance is analyzed based on gene expression and clinical information, and their association with PTC in the current state of knowledge is evaluated individually and as a whole. R-scripts corresponding to this pipeline are available at our GitHub repository (https://github.com/icic-uns-conicet/PTC-GeneExpAnalysis.git (accessed on 30 May 2023)).

## 2. Data and Methods

### 2.1. Data

The analysis starts with a thorough exploration of three datasets: GSE29265 [11], GSE33630 [12,13] and the TCGA-THCA project [10]. The first and second correspond to array expression profiling (microarray) and the last one to RNA-seq. Concerning the samples’ distribution in each dataset, the initial configuration is shown in Table 1. Moreover, regarding clinical and phenotypic information, four tables are provided as Supplementary Material (see “GSE33630.csv”, “GSE29265.csv”, “TCGA THCA survival.tsv” and “TCGA THCA penotype.csv” for more details).

For the case of GEO datasets, even though they belong to works carried out by the same authors, they report different experiments, so it is relevant to specify that there is no overlap between the samples in GSE33630 and GSE29265. It is important to remark that only some extra information is available for dataset GSE33630 apart from the pathological diagnostic, namely the tissue type field, corresponding to tumour or patient-matched non-tumour and title field which maps PTC, ATC and normal. On the other hand, a few more data are given for GSE29265; origin field for sporadic or Chernobyl, characteristics field for height and weight of the tumour, Braf field with positive and negative values, age at incident and age at operation fields, among others. This does not hold for all the samples. On the contrary, it is only complete in a very low percentage of them.

From these datasets’ configurations of samples, a design decision of our research was to perform this entire study considering, within the tumor cases, only PTC. As previously mentioned, ATC samples were analyzed in a previous work, in which we were able to obtain a great amount of information regarding relevant genes for ATC in the study of thyroid cancer [7]. In this context, in the present work, we seek to discover new genes associated with the other and more common variant of cancer, PTC. For this aim, we performed several filtering procedures on the groups of samples of each dataset, yielding the following final structure: GSE33630, 49 PTC and 45 control; GSE29265, 20 PTC, and 20 control; TCGA-THCA, 457 PTC (Papillary adenocarcinoma and Papillary carcinoma) and 55 control. It is important to remark that control samples, in most cases, correspond to healthy tissues adjacent to the tumors.

Respecting the samples that were kept and the samples that were deleted from the study, we provide 9 files with the corresponding samples ID. The name of the file indicates whether the sample was kept (IN) or deleted (OUT).

Regarding the presence of noise in the counts’ matrix, all the datasets were filtered and cleaned carrying out traditional pre-processing procedures. Genes with expression profiles that contained a high amount of outliers, the majority of zeros and/or minimal variance, were removed from the study.

### 2.2. Methods

Statistical learning is an essential discipline in any bioinformatic study. For the first stage of this work, the three datasets are analyzed independently with a univariate statistical approach. For this, differentially expressed genes are detected in different manners depending on whether the data come from microarray or RNA-seq experiments. For the former, which are those obtained in GEO, the R package limma [14] was used. A linear model is fitted with the expression data, the corresponding t-test or F-test statistics are computed and log-odds of the differential expression are obtained by empirical Bayes moderation of the standard errors until a final value is obtained. The result of this step is a list of differentially expressed genes, which fold change allows distinguishing between those that are up-regulated and those that are down-regulated in PTC. In the case of TCGA data, the edgeR package [15] was used. In this case, differential expression analysis is implemented using the exact test proposed by Robinson and Smyth [16] to obtain a mean difference between the two groups of negative binomial random variables under study. Given the huge amount of tests performed over the expression matrices, all the results presented in this paper are based on adjusted p-values, using Benjamini–Hochberg to correct multiple-testing error [17]. The same thresholds were used for the three assays: 0.01 for the adjusted p-value and 2 for the fold-change.

Secondly, the Recursive Feature Elimination (RFE) algorithm [18] is applied to the chosen genes, thus yielding a final group of four genes of interest. RFE is one of the most widely used algorithms for feature selection, and it showed excellent performance for gene expression data in the previous work [7]. The idea behind this method is to reduce the complexity of the model by eliminating genes one by one until the optimal amount is left. It is one of the most popular feature selection algorithms due to its flexibility and ease of use. Two hyperparameters are important to define when using RFE: the first is the maximum number of genes to select, and the second is the algorithm used to help choose those genes. In our case, we put no limit on the number of genes and used Random Forest as the supporting algorithm.

Next, a punctual observation is performed individually for each gene to verify their association with thyroid cancer. First, box plots were graphed to illustrate their differential expression and Kaplan–Meier plots were built from clinical data to validate the relevance of these genes in the survival analysis context. The main parameters were set as follows: patients were split by the median, the best cut-off was automatically selected, survival analysis corresponds to the feature Overall Survival (OS) and the follow-up threshold includes all amounts of months. In this way, a Kaplan–Meier plot was obtained for each selected gene, including the statistical significance according to its corresponding *p*-value.

Moreover, relapse-free survival (RFS) constitutes an important measure to be validated regarding the putative influence that a gene (or its expression level) might have over it. In cancer, RFS is the length of time after primary treatment for cancer ends that the patient survives without any signs or symptoms of that cancer. Therefore, we also performed this analysis, with the same web tool and configuration as aforementioned for OS. In addition, several clinical variables were analyzed to corroborate their association with the expression profiles of the 4 genes. Dependence was assessed individually, and in combination with a linear model.

Finally, a hand-made literature search of previous studies relating each of these genes individually to thyroid cancer was performed, and a protein–protein interaction network was generated using STRING [19,20] to analyze the relationship of the group of genes as a whole based on several sources of knowledge. The settings for building the network were as follows:

- Network type: full STRING network (the edges indicate both functional and physical protein associations).
- Meaning of network edges: evidence (line color indicates the type of interaction evidence).
- Active interaction sources: Text mining, Experiments, Databases, Co-expression, Neighborhood, Gene Fusion, Co-occurrence.
- Minimum required interaction score: 0.4.
- Max number of interactors to show:
  - 1st shell: no more than 5.
  - 2nd shell: no more than 5.

## 3. Results

**Figure 1:**
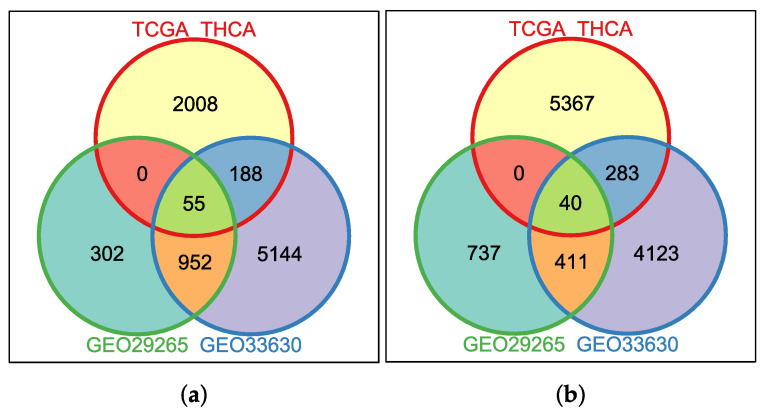
DEGs found for each dataset: (**a**) Up-regulated in PTC; (**b**) Down-regulated in PTC.

Three datasets (GSE33630, GSE29265 and TCGA-THCA) were individually analyzed to identify differentially expressed genes (DEGs) in unpaired cancerous and non-cancerous thyroid tissues. The numbers of DEGs in each case, and the proportion of up-regulated genes in tumor tissues, are shown in Table 2. Overlaps between the DEGs obtained from each of the datasets can be observed in Figure 1. The 95 genes that constitute the intersection of the three sets were separated in each case into 55 for up-regulated and 40 for down-regulated in PTC (Table 3).

**Figure 2:**
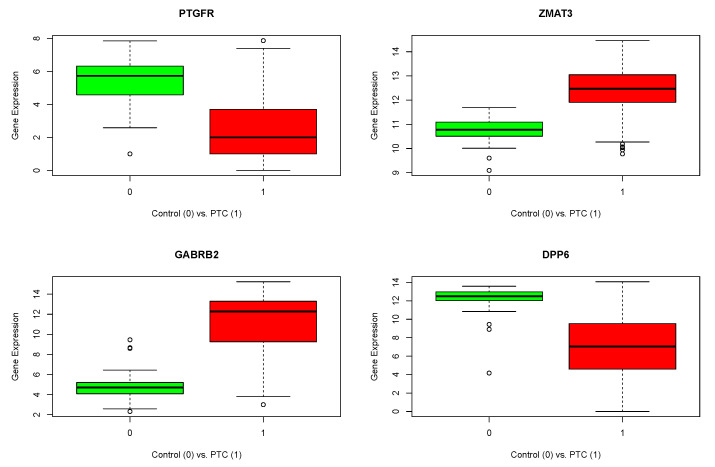
Box plots assessing differential expression behavior of genes of interest (color green for normal samples and red for tumor samples).

In the second stage of exploration, we reduced the number of rows of the TCGA expression matrix by using these 95 genes. At this point, the decision was to continue with the RNA-seq dataset alone since it has the largest number of samples (457 cases). This scenario made it possible to resample a given amount of PTC samples to perform several runs using different data as follows. With the sub-matrix, we proceeded to apply RFE repeatedly using 55 original control samples, and 55 PTC samples that changed between each run. Since the RFE algorithm is non-deterministic, the combination of repeated runs with different input data helped in obtaining more robust and accurate results. Fifty runs of the algorithm were performed, each one with automatic tuning of the number of features selected with 10 cross-validations. Each run returned an output list of selected genes. Finally, the genes that appeared in the intersection of the lists returned by all the runs are indicated as genes of interest. In order of importance according to the RFE algorithm, these genes are *PTGFR*, *ZMAT3*, *GABRB2* and *DPP6*. The following box plots were generated to illustrate their differential expression (Figure 2). In Table 4, the details of adjusted *p*-values and fold changes for the four selected genes are reported.

In the following step, a study of the relation between clinical and expression data was performed employing an online tool that generates survival plots [21,22]. Pan-cancer RNA-seq section was used, where 507 tumor samples are available for thyroid carcinoma. In the Table 5, a summary of clinical data is presented [22].

**Figure 3:**
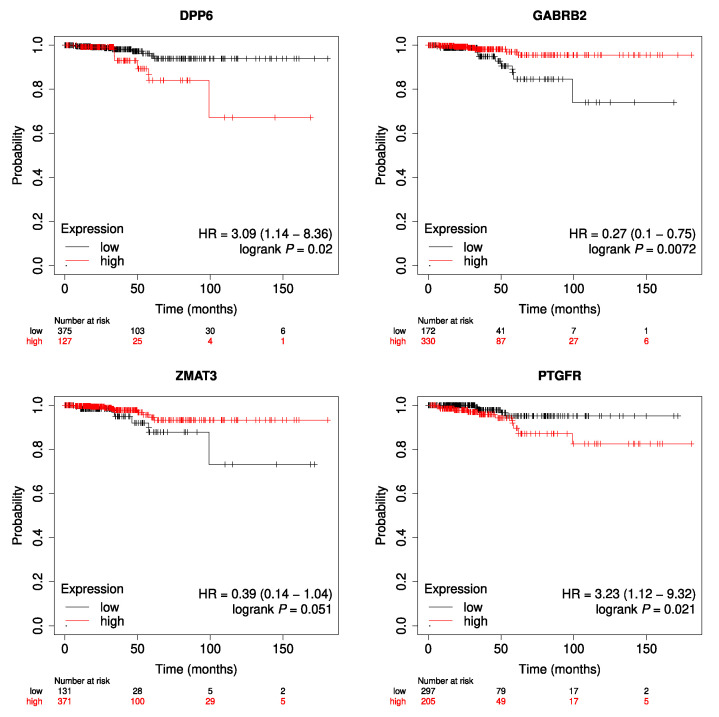
Kaplan–Meier Overall Survival plots.

**Figure 4:**
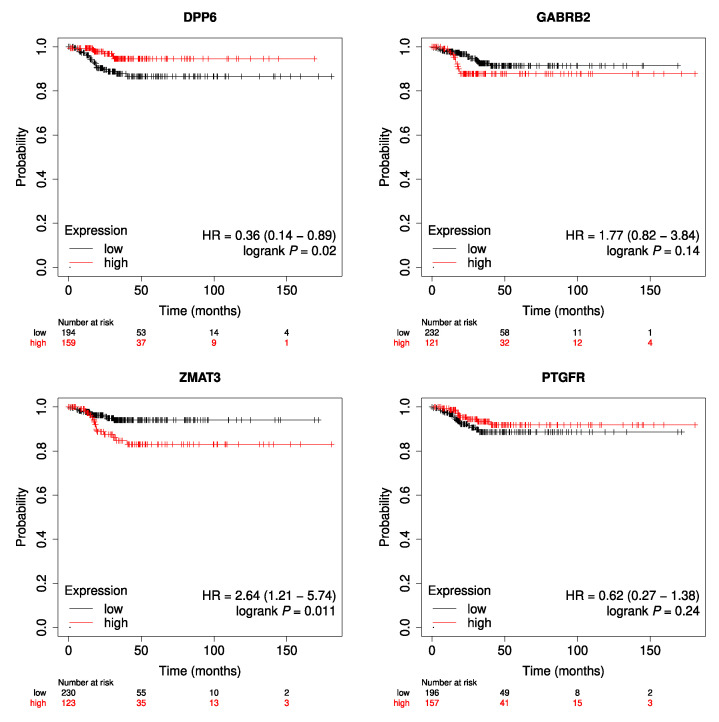
Kaplan–Meier Relapse-Free Survival plots.

In order to mention some specific values regarding clinical data, all the tumor samples correspond to the thyroid as the primary site; 135 are male cases, and 367 are females. Up to the date this text is being written, 16 cases died and 486 are still alive. Figure 3 shows the Kaplan–Meier plots for each of the genes, all with *p*-values indicating the statistical significance. On the other hand, Figure 4 illustrates the results obtained for RFS analysis for each of the genes.

As an ultimate analysis stage for clinical data, an association assessment was performed between some clinical variables and the expression profiles of the four genes of interest: PTGFR, ZMAT3, GABRB2 and DPP6. Kendall non-parametric correlation was calculated for the numeric variable, and Anova tests were performed to determine the extent to which categorical variables explain the expression profile of each gene. The clinical variables analyzed are as follows:

- Age at initial pathologic diagnosis
- Gender
- Race
- Primary thyroid gland neoplasm location
- Tumor stage

It is important to observe that, for this data analysis, the samples that were used are those that are common with the expression matrix. This slightly changes the configuration of samples from the one presented in Table 5, where information of the samples used by the KM plot web tool was reported. In this case, age at initial diagnosis varies from 15 to 88 years old, with a mean of 46.74. Gender is stored as male and female consisting of 122 and 335 cases respectively. Races are: black or African American, white, American Indian or Alaska native, Asian and not reported; consisting of 25, 297, 1, 46 and 88 cases respectively. Primary location values are: not reported, bilateral, isthmus, left lobe and right lobe; consisting of 6, 74, 19, 159 and 199 cases respectively. Tumor stages are: not reported, stage i, stage ii, stage iii and stage iv; consisting of 2, 267, 51, 90 and 47 respectively. In Table 6 we summarize the results of all associations’ assessments. Correlation between AGE at initial diagnosis and the expression profile of each gene of interest is reported by the pair: *p*-value/Tau value. The association of each categorical clinical variable with the expression profile of each gene of interest is reported through the pair: *p*-value/F value.

From the results we can conclude that there is no correlation with the age for any of the genes’ expression profiles. Furthermore, no association is evidenced with gender for any of the genes. Regarding the race, it is detected that it might have some influence in the expression of GABRB2 and PTGFR. Considering tumor location, it is observed that it might be associated to the expression profile of DPP6. Apropos tumor stage, it is seen that GABRB2, DPP6 and PTGFR might have some relation with the progression of the disease. Finally, one model including the five clinical variables to explain the expression profile of each gene was assembled. Results are shown in Table 7.

From these results, with the four models combining all the variables as predictors, it can be concluded that DPP6 and GABRB2 are the genes whose expression profiles are better explained by the majority of the clinical variables. As a general rule, the gender does not contribute significantly to any of the models.

**Figure 5:**
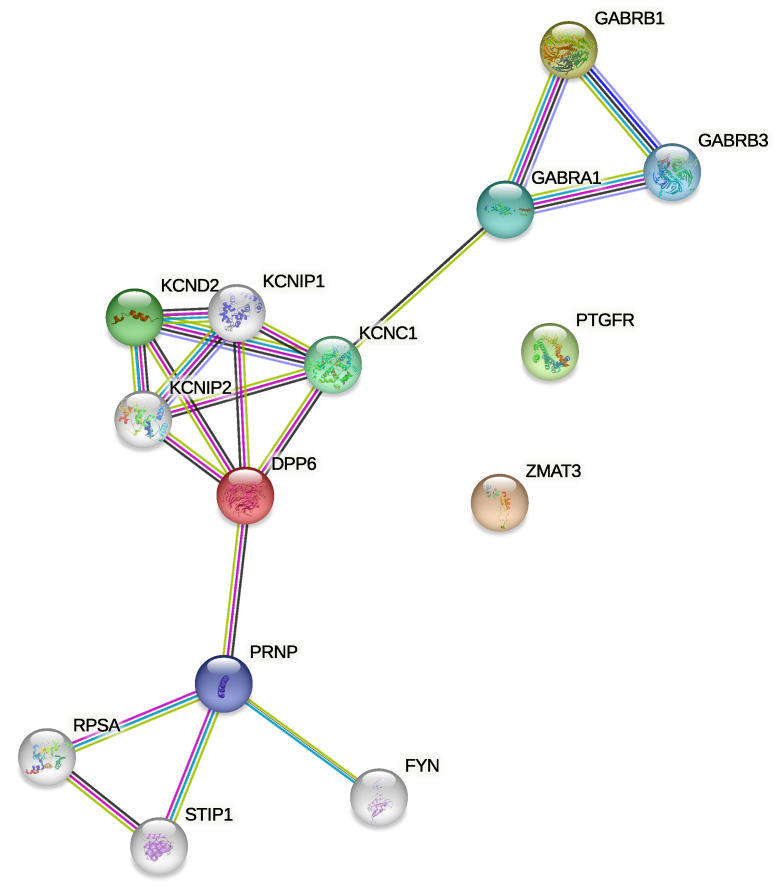
PPI network for *PTGFR*, *ZMAT3*, *GABRB2* and *DPP6*.

Furthermore, a hand-made literature review was performed to detect previous evidence of associations between these genes and thyroid cancer. Then, we searched for associations between the four genes as a cluster, building a Protein–Protein Interaction (PPI) network with the STRING tool [19,20]. This tool constitutes a means of comprehensively and automatically finding associations between proteins (or as in this case, their corresponding genes) that may appear in different knowledge storage sources, such as PUBMED articles, KEGG Pathways, GO terms and Reactome, among others. The PPI network is shown in Figure 5.

As a result, a PPI network was built with an enrichment *p*-value of 3.23 × 10${}^{-5}$. Together with the network, the tool provides a table with all the information used to obtain the interactions, supplied as Supplementary Material (see String.csv).

Finally, the gene set of connected proteins composed of *DPP6, GABRB2, RHOT1, TRAK2, GABRB3, GABRA1, GABRG2, KCNC1, KCND2, KCNIP3, KCNIP1* and *KCNIP2* was used for a gene set enrichment analysis. Enrichment tables constructed for this group of genes regarding GO molecular function, GO biological process, Kegg and Reactome pathways, and PheWeb and DisGeNET were consulted. Uniquely for DisGeNET, PTC is found to have some association with the group of genes. DisGeNET contains collections of genes and variants associated with human diseases. The complete enrichment table obtained for DisGeNET is provided as Supplementary Material (see DisGeNET.txt).

## 4. Discussion

During the first stage of this research work, the hypothesis was to verify the consistency between the differential expression information obtained from microarray experiments to the one inferred from RNA-Seq experiments. As can be seen in Figure 1, if we focus on the two GEO experiments, we can see that the amount of genes in common is much higher than the amount they share with those obtained from TCGA. However, there are also genes in common between the three datasets, which leads us to think that it might be necessary, when we are looking for highly representative genes, to perform at least two filtering stages to reach those genes that seem to be the most relevant for the distinction between the samples’ groups.

Another issue that comes up in Figure 1 is that the proportion of DEGs in TCGA is considerably smaller than in the first two cases. In this regard, we should not lose sight of a very relevant factor for the samples labeled as “control” in the case of RNA-seq data. Here, samples from the TCGA-THCA labeled as Solid Normal Tissue do not correspond to healthy individuals. They correspond to “healthy” tissue adjacent to the tumor. In this sense, several studies have shown that this characteristic of control samples can affect the results of this type of analysis. It is a better practice to use tissue from the GTEx project for control samples, where tissue measurements from different organs of healthy patients are stored. The problem is that integration of TCGA data with GTEx is not a trivial task, and it requires a careful analysis of how expression values are measured, stored and normalized in each case. Consequently, all the integration procedure is left as future work. All in all, an interesting group of DEGs in common was found, the 95 genes of interest, of which 55 are up-regulated and 40 are down-regulated in PTC.

Using those 95 genes, the second core step of the pipeline was performed, where a second filtering task took place using the TCGA counts matrix. First, the expression matrix was reduced, leaving 95 rows corresponding to the genes of interest. With this new sub-matrix, RFE was repeatedly executed, until a new and final subset of genes of interest was found: *PTGFR*, *ZMAT3*, *GABRB2* and *DPP6*. At this point of the analysis, it is important to remark that an enrichment analysis of the 95 genes was carried out for KEGG pathways and GO biological process. No GO term or KEGG pathway was enriched by that gene cluster. This means that these genes (or a subset of them) have never been found together associated with the same metabolic pathway or the same biological process.

In Figure 2, box plots show that the four genes of interest exhibit a perfectly differentiated behavior between control and tumor sample groups. As mentioned earlier, it was found that *GABRB2* and *ZMAT3* mRNA expressions are up-regulated in PTC compared to tissue adjacent to the tumor, and that *GABRB2* mRNA expression is associated with the best survival in PTC, suggesting its potential usefulness as a prognosis marker. *GABRB2* (γ-aminobutyric acid (GABA) A receptor, β2) is a gene located at the human chromosome 5q34 and encodes the β2 subunit of the *GABAA* receptor, which mediates the inhibitory effect of *GABA* in the central nervous system. In accordance with our results, other authors identified *GABRB2* as a diagnostic marker of malignant thyroid lesions [23,24]. It was also demonstrated that *GABRB2* mRNA expression positively correlates with lymph nodes, and it is relevant for the proliferation, migration, invasion and apoptosis of human thyroid cancer cell lines [25]. In addition, *GABRB2* was found to be regulated by DNA methylation and postulated as a potential driver of PTC development [25,26]. *ZMAT3* (zinc finger matrin-type 3) is a gene located at the human chromosome 3q26 that encodes a zinc finger protein that localizes in the nucleus and is induced by wild-type p53 expression, playing a role in the p53-dependent growth regulatory pathway. Of note, *ZMAT3* induction by the erytropoietin/erytropoietin receptor (Epo/EpoR) axis was reported in the papillary thyroid cancer subtype but not in others as anaplastic and follicular thyroid cancer [27]. Regarding *PTGFR* and *DPP6*, to our knowledge, both genes fail to be associated with thyroid cancer to date.

Likewise, the relevance of the genes in thyroid cancer can be observed in the subsequent step, for Overall Survival and Relapse-Free Survival analysis. OS plots show that all genes have a correlation between their expression values and the clinical data, *GABRB2* being the most meaningful one. This can be appreciated in the significant curves and small *p*-values of Kaplan–Meier plots. Furthermore, regarding RFS analysis, even for *GABRB2* and *PTGFR* the *p*-values are not significant. For the cases of genes *ZMAT3* and *DPP6*, *p*-values are less than 0.05 thus showing that they have some relevant association with this metric. It is important to remark that, for the Kaplan–Meier plots, only tumor samples are considered. So, what is observed in the curves is the difference in the expression of cancer tissues regarding overall survival. In this case, control samples are never used for the illustration. What is evident from the plots is that higher or lower values of expression of the genes under study drastically change the survival prognostic of the individuals. Furthermore, from the analysis of associations between gene expression profiles and clinical variables, it could be observed that there are interesting dependencies both individually, and also in a multivariate approach. Mainly, the expression profiles of DPP6 and GABRB2 in tumor cases seem to be clearly related with most of the clinical variables under study.

Finally, after having verified that the genes may be relevant for thyroid cancer according to expression data, and having checked individually whether they had already been associated with the disease or not, we made a comprehensive evaluation of the four genes in terms of the interaction of the associated proteins. This was done with the STRING tool that searches numerous sources for the occurrence of associations, and the network shown in Figure 5 was constructed. To construct the network, we set the tool to find relationships with a maximum of five indirect interactions. The first thing we can observe is that some associations were found between the *DPP6* and *GABRB2* genes. However, *PTGFR* and *ZMAT3* genes have no edges connecting them in any way with the rest of the nodes. This makes them more interesting genes to be investigated in depth. Furthermore, if we look at the table that is generated in STRING as output (in addition to the graph) with the details of the interactions and the sources from which they were inferred, it is seen that *GABRB2* and *DPP6* were reported 231 times as co-expressed genes. In the output table, the details of all the other genes that are part of the network can be observed, information that in turn could be interesting to find other potential genes linked to PTC. This result can be easily reproduced, by querying STRING to build the network for the four genes of interest, setting the parameters as explained below.

## 5. Conclusions

As a result of this work, four putative gene markers for Papillary Thyroid Cancer were discovered. Three gene expression datasets were analyzed: two from microarray experiments and one from RNA-seq. The aim was to detect new genes relevant in the comparison between control and PTC samples. Two consecutive phases of differential gene expression filtering were performed: the first one was implemented with univariate statistics; the second one was carried out by means of the machine learning algorithm RFE. As a result of the first stage, a subset of 95 genes that matched in the 3 datasets was obtained, 55 up-regulated and 40 down-regulated in PTC. After a second step of feature selection with RFE performed on the 95 genes, 4 potential genes of interest were found which proved to be very relevant in the differentiation between healthy tissue and PTC, and that have not yet been significantly associated with the disease. The potential marker genes found in this work are *PTGFR*, *ZMAT3*, *GABRB2* and *DPP6*. After an insightful literature search, we can conclude that the most relevant and promising ones to be studied in more depth are *PTGFR* and *DPP6*. It remains future work to assess these results using GTEx as a main source of healthy samples, integrating them with TCGA tumor samples.

## Figures and Tables

**Table 1:** Summary of used datasets.

	Sample Distribution	Source	Details
GSE33630	11 anaplastic thyroid carcinomas (ATC), 49 papillary thyroid carcinomas (PTC) and 45 normal thyroids (N)	GEO	https://www.ncbi.nlm.nih.gov/geo/query/acc.cgi?acc=gse33630 (accessed on 30 May 2023)
GSE29265	9 anaplastic thyroid carcinomas (ATC), 20 papillary thyroid carcinomas (PTC) and 20 normal thyroids (N)	GEO	https://www.ncbi.nlm.nih.gov/geo/query/acc.cgi?acc=gse29265 (accessed on 30 May 2023)
TCGA-THCA	1 Carcinoma, 1 Follicular adenocarcinoma, 1 Follicular carcinoma, minimally invasive, 4 Non encapsulated sclerosing carcinomas, 1 Oxyphilic adenocarcinoma, 444 Papillary adenocarcinomas, 43 Papillary carcinomas, columnar cell, 120 Papillary carcinomas, follicular variant	TCGA	https://www.cancer.gov/types/thyroid (accessed on 30 May 2023) https://xenabrowser.net/datapages/?cohort=GDC%20TCGA%20Thyroid%20Cancer%20(THCA)&removeHub=https%3A%2F%2Fxena.treehouse.gi.ucsc.edu%3A443 (accessed on 30 May 2023)

**Table 2:** Differential expression analysis.

	Assesed Genes	DEGs	Upregulated in PTC		Downregulated in PTC	
GSE33630	23,521	11,196	6339	57%	4857	43%
GSE29265	23,521	2497	1309	53%	1188	47%
TCGA-THCA	60,488	7941	2251	28%	5690	72%
Overlap	19,516 genes in common between the 3 assays					

**Table 3:** Genes (gene symbol IDs) up and down-regulated in PTC.

Expression in PTC	DEGs (Gene Symbol)
Up	*WNT4, EDARADD, MYT1L, ZNF385B, B3GNT7, ARPP21, RP11-757F18.5, WDR49, ZMAT3, LPP-AS2, FAM43A, PCDH10, C4orf45, GALNTL6, ARHGEF28, SPINK6, C5orf47, LY86-AS1, PDE1C, KCP, ASB10, ERICH1-AS1, TDH, PSKH2, C8orf88, C9orf66, TRPM3, ATOH7, SYCE1, SYT9, GLYATL2, TYR, FLJ12825, NAV3, FAM124A, ABCC4, RPS6KA5, LINC00521, ASPG, CYP19A1, PRR35, ASPHD1, CNTNAP4, LINC00304, TRPV3, CD300LG, RP11-700H6.1, TK1, NETO1, ZNF99, GABRB2, SIGLEC11, KLK4, ZSCAN4, CBS*
Down	*NEXN, PTGFR, RP11-498C9.17, AMER3, FN1, ADAMTS9, SLC7A14, GPR111, TAAR1, DPP6, RSPO2, SLC30A8, IFNE, TMEM252, AKR1E2, C10orf107, C10orf55, TECTB, PLEKHS1, SLC18A2, THRSP, PIANP, KRT73, C12orf74, FAM71C, RFX4, MYO16, RPPH1, GSC, NOX5, GAS2L2, B4GALNT2, STARD6, ZNF560, KLK8, NLRP11, PAX1, WFDC11, LKAAEAR1, FMR1NB*

**Table 4:** Fold Change values and adjusted *p*-values for genes of interest.

	ENSID	Gen Symbol	logFC	logCPM	*p* Value	FDR
1	ENSG00000122420.8	*PTGFR*	−0.89475	13.48313	2.37 $\times \;10^{-20}$	2.25 $\times \;10^{-19}$
2	ENSG00000130226.15	*DPP6*	−0.56845	14.50570	9.07 $\times \;10^{-20}$	7.18 $\times \;10^{-19}$
3	ENSG00000145864.11	*GABRB2*	1.42467	14.88948	1.98 $\times \;10^{-77}$	1.88 $\times \;10^{-75}$
4	ENSG00000172667.9	*ZMAT3*	0.40854	15.06364	7.30 $\times \;10^{-12}$	2.77 $\times \;10^{-11}$

**Table 5:** Summary of clinical data for available samples for thyroid carcinoma.

Tumor type	Thyroid
TCGA code	THCA
Samples with RNA-seq data	502
Median survival–OS (months)	31.47
Events (n)	16
Median survival time in patients with an OS event	34.03
Median survival–RFS (months)	18.72
Median survival in patients with a relapse (months)	16.43
Sex (F/M)	367/135
Stage (S0/S1/S2/S3/S4)	0/281/52/112/55
Grade (low/high)	–
Race (White/Asian/Black-African)	332/51/27

**Table 6:** Independent associations between expression profiles and clinical variables. The numbers highlighted in bold correspond to the values obtained for variables that were found to be correlated with the expression value.

	ZMAT3	GABRB2	DPP6	PTGFR
AGE at initial diagnosis	0.001439/−0.100624	0.000313/−0.113823	0.378/0.027890	0.7956/−0.008471
Gender	0.688/0.162	0.848/0.037	0.635/0.225	0.159/1.986
Race	0.592/0.7	**0.00033/5.338**	0.0845/2.064	**0.00632/3.631**
Primary thyroid gland neoplasm location	0.191/1.535	0.0619/2.26	**0.00776/3.511**	0.148/1.705
Tumor stage	0.221/1.436	**0.000372/5.269**	**0.00103/4.687**	**0.00168/4.401**

**Table 7:** Models explaining gene expression profile by assembling clinical features as independent variables.

ZMAT3	Df	Sum Sq	Mean Sq	F value	Pr(>F)	
Tumor stage	4	4.14	1034	1463	0.21251	
Gender	1	0.12	0.123	0.174	0.67681	
Primary Location	4	4.25	1063	1503	0.20026	
Race	4	1.88	0.470	0.665	0.61672	
AGE at initial d.	1	6.88	6878	9729	0.00193	**
Residuals	442	312.50	0.707			
GABRB2	Df	Sum Sq	Mean Sq	F value	Pr(>F)	
Tumor stage	4	138.1	34.54	5660	0.000189	***
Gender	1	2.0	2.02	0.331	0.565269	
Primary Location	4	57.9	14.46	2370	0.051792	.
Race	4	125.5	31.38	5142	0.000466	***
AGE at initial d.	1	80.4	80.41	13,178	0.000316	***
Residuals	442	2697.0	6.10			
DPP6	Df	Sum Sq	Mean Sq	F value	Pr(>F)	
Tumor stage	4	172	43.01	4965	0.000636	***
Gender	1	0	0.19	0.022	0.882296	
Primary Location	4	163	40.71	4700	0.001007	**
Race	4	89	22.36	2582	0.036718	*
AGE at initial d.	1	66	65.83	7599	0.006083	**
Residuals	442	3829	8.66			
PTGFR	Df	Sum Sq	Mean Sq	F value	Pr(>F)	
Tumor stage	4	61.8	15,454	4509	0.0014 *	*
Gender	1	8.6	8613	2513	0.1136	
Primary Location	4	22.9	5736	1673	0.1551	**
Race	4	39.1	9774	2852	0.0235 *	*
AGE at initial d.	1	1.6	1604	0.468	0.4942	**
Residuals	442	1515.0	3428			
Signif. codes:	0 ‘***’	0.001 ‘**’	0.01 ‘*’	0.05 ‘.’	0.1 ‘ ’	1

## Data Availability

Raw files of the first two datasets used in this work can be found at GEO [8,9] by using the corresponding accession numbers: GSE29265 (https://www.ncbi.nlm.nih.gov/geo/query/acc.cgi?acc=GSE29265 (accessed on 30 May 2023)) and GSE33630 (https://www.ncbi.nlm.nih.gov/geo/query/acc.cgi?acc=GSE33630 (accessed on 30 May 2023)). The dataset corresponding to the TCGA-THCA project [10] can be found at https://xenabrowser.net/datapages/?cohort=GDC%20TCGA%20Thyroid%20Cancer%20(THCA)&removeHub=https%3A%2F%2Fxena.treehouse.gi.ucsc.edu%3A443 (accessed on 30 May 2023).

## Supplementary Materials

The supplementary material can be downloaded from: https://github.com/icic-uns-conicet/PTC-GeneExpAnalysis.git (accessed on 30 May 2023). All supplementary material have been uploaded to GitHub.

## Abbreviations

The following abbreviations are used in this manuscript:

ATC	Anaplastic Thyroid Carcinomas
cDNA	complementary DNA
DEGs	Differentially Expressed Genes
GEO	Gene Expression Omnibus
GO	Gene Ontology
GTEx	Genotype-Tissue Expression
KEGG	Kyoto Encyclopedia of Genes and Genomes
KM	Kaplan–Meier
OS	Overall Survival
PCR	Polymerase Chain Reaction
PPI	Protein–Protein Interaction
PTC	Papillary Thyroid Carcinoma
RFE	Recursive Feature Elimination
RFS	Relapse Free Survival
RNA-seq	RNA sequencing
TCGA	the Cancer Genome Atlas
TCGA-THCA	The Cancer Genome Atlas Thyroid Cancer
